# Systematic mapping of checklists for assessing transferability

**DOI:** 10.1186/s13643-018-0893-4

**Published:** 2019-01-14

**Authors:** Heather Munthe-Kaas, Heid Nøkleby, Lien Nguyen

**Affiliations:** Norwegian Institute of Public Health, Oslo, Norway

**Keywords:** Transferability, Indirectness, Relevance, Evidence, Systematic review, Applicability

## Abstract

**Background:**

Systematic reviews of research evidence have become an expected basis for decisions about practice guidelines and policy decisions in the health and welfare sectors. Review authors define inclusion criteria to help them determine which studies to search for and include in their reviews. However, these studies may still vary in the extent to which they reflect the context of interest in the review question. While most review authors would agree that systematic reviews should be relevant and useful for decision makers, there appears to be few well known, if any, established methods for supporting review authors to assess the transferability of review findings to the context of interest in the review. With this systematic mapping and content analysis, we aim to identify whether there exists checklists to support review authors in considering transferability early in the systematic review process. The secondary aim was to develop a comprehensive list of factors that influence transferability as discussed in existing checklists.

**Methods:**

We conducted a systematic mapping of checklists and performed a content analysis of the checklist criteria included in the identified checklists. In June 2016, we conducted a systematic search of eight databases to identify checklists to assess transferability of findings from primary or secondary research, without limitations related to publication type, status, language, or date. We also conducted a gray literature search and searched the EQUATOR repository of checklists for any relevant document. We used search terms such as modified versions of the terms “transferability,” “applicability,” “generalizability,” etc. and “checklist,” “guideline,” “tool,” “criteria,” etc. We did not include papers that discussed transferability at a theoretical level or checklists to assess the transferability of guidelines to local contexts.

**Results:**

Our search resulted in 11,752 titles which were screened independently by two review authors. The 101 articles which were considered potentially relevant were subsequently read by two authors, independently in full text and assessed for inclusion. We identified 31 relevant checklists. Six of these examined transferability of economic evaluations, and 25 examined transferability of primary or secondary research findings in health (*n* = 23) or social welfare (*n* = 2). The content analysis is based on the 25 health and social welfare checklists. We identified seven themes under which we grouped categories of checklist criteria: population, intervention, implementation context (immediate), comparison intervention, outcomes, environmental context, and researcher conduct.

**Conclusions:**

We identified a variety of checklists intended to support end users (researchers, review authors, practitioners, etc.) to assess transferability or related concepts. While four of these checklists are intended for use in systematic reviews of effectiveness, we found no checklists for qualitative evidence syntheses or for the field of social welfare practice or policy. Furthermore, none of the identified checklists for review authors included guidance to on how to assess transferability, or present assessments in a systematic review. The results of the content analysis can serve as the basis for developing a comprehensive list of factors to be used in an approach to support review authors in systematically and transparently considering transferability from the beginning of the review process.

**Electronic supplementary material:**

The online version of this article (10.1186/s13643-018-0893-4) contains supplementary material, which is available to authorized users.

## Background

Evidence-based decision making has become a common ideal within healthcare, and to a lesser degree within social welfare. Increasingly, systematic reviews of research evidence have become an expected basis for decisions about practice guidelines and policy decisions in these sectors. Much research, discussion and thought has gone into developing and improving evidence synthesis methods, most notably by organizations such as the Cochrane and Campbell Collaborations and particularly with regard to questions of intervention effectiveness [1, 2]. While methods for synthesis are far from fully developed, they appear to have matured to a position where the focus is shifting to also include discussion on, and development of methods for, improving the usefulness of evidence from systematic reviews for decision makers [3–7].

Despite this movement toward increased consideration of the relevance of review findings to decision making contexts, there is currently no consensus on how to systematically and transparently consider and assess factors that may influence the transferability of review findings and present such assessments to decision makers. For the purpose of this paper, the terms *decision makers* and *end users* broadly refer to individuals or groups who may use findings from a systematic review and may include policymakers, practitioners, or policy analysts [5]. Furthermore, we define transferability as whether the level of effectiveness (or perceptions and experiences) of an intervention in a specific setting or population will be similar to the observed level of effectiveness (or perceptions and experiences) observed in a systematic review ([8] as cited in [9]). Other terms related to transferability include applicability, generalizability, transportability, directness, extrapolation, internal/external validity, and relevance and are discussed at length elsewhere [9–11]. In particular, Burford and colleagues provide a useful overview of the most commonly used terms and their definitions (see Table 1 below) [9]. We have adapted the definition of “transferability” that is presented in Table 1.

**Table 1:** Definitions of transferability and related terms in the context of systematic reviews of effects

Term	Definition
Transferability	Whether when implementing an intervention in a particular setting or population, the level of effectiveness of the intervention (i.e., the effect size) will be similar to that observed in the systematic review. Both absolute and relative effects should be considered.
Applicability	Whether the findings of a review can be applied in a particular context or population. This includes consideration of the feasibility of implementing the intervention and variation in intervention fidelity, population characteristics, context, culture, values, and preferences.
Directness	One of five criteria in the Grading of Recommendations Assessment, Development and Evaluation (GRADE) framework for assessing the overall quality of a body of evidence. Four types of directness (or indirectness) are considered: differences between the (1) population, (2) intervention or (3) outcomes of interest and those in studies, and (4) indirect comparisons (i.e., when there are no studies directly comparing two or more interventions of interest, and authors compare those interventions indirectly using evidence from different studies).
External validity	The extent to which results provide a correct basis for generalizations to other circumstances. For instance, a meta-analysis of trials of elderly patients may not be generalizable to children.
Extrapolation	The process of generalizing results to circumstances beyond the original observations. Also see external validity
Generalizability	See external validity.
Internal validity	The extent to which a review has minimized potential sources of bias and, in doing so, answered the review question “correctly.”

Adapted from Burford [9]

### Methods for improving evidence usefulness

Evidence-informed decision making is not without its challenges and limits [12]. One such challenge is that widespread use of evidence-informed decision making may lead to overly “rule-based” practice, where context and individual clients’ needs are not adequately considered [12]. Greenhalgh and colleagues have suggested that “[p]roducers of evidence summaries, clinical guidelines, and decision support tools must take account of who will use them, for what purposes, and under what constraints” [12] (p.4). Simply synthesizing the range of primary studies is necessary but not sufficient to ensure evidence-based decision making: “an important and additional necessary step is adaptation […] to the context of use” [13](p. 111). The context of interest in a review is usually specified by defining, for example, the population, intervention, comparison, and outcomes of interest. An example of such may be the effect of an employment program compared to usual services on days in paid employment for adults with mental illness. However, the findings from this review may be intended for use in a specified local context (e.g., a country in Scandinavia) where factors related to hiring practice and the welfare system differ substantially from contexts (such as USA) where the many of the included studies come from. These factors are not specified in the review question, but may influence the transferability of the review findings to the context of interest in the review [14].

### Considering context in the systematic review process

Systematic review authors are often encouraged to consider context and factors that may influence applicability of the review findings. Cochrane requires a discussion of applicability of the evidence in systematic reviews [1]. The Grading of Recommendations Assessment, Development, and Evaluation (GRADE) approach for evidence of effectiveness and the GRADE-CERQual approach for qualitative evidence are each designed to assess confidence in findings from evidence syntheses. Both approaches include an assessment of *indirectness* or *relevance* of the evidence to the review question [15, 16]. However, neither approach provides specific guidance on how review authors should transparently and systematically make assessments for these components. Currently, systematic review authors are often left to make an ad hoc assessment of *indirectness* or *relevance* which is not clearly transparent to the end user. A potential risk of this is that the decision maker may, often unconsciously, reduce their certainty or confidence in a review finding based on their own assessment of the relevance of the included studies to the context of interest. This can result in the evidence being downgraded by both the review author and the decision maker, thereby presenting an overly negative assessment of certainty or confidence. Conversely, the review author may downgrade for indirectness or relevance based on factors that the decision maker does not consider relevant to the transferability of the findings, thereby also misrepresenting an assessment of certainty or confidence that is skewed toward the negative. A more transparent approach for considering transferability of review findings could help to assuage the above issues.

### Previous research

As the focus on transferability, applicability, and generalizability of research increases, so does the number of tools for assessing these concepts. These tools vary significantly in terms of the terminology they use and how they define the concepts they aim to address, the audience and the thematic area. Some tools aim to assess external validity of primary studies (e.g., Dekkers and colleagues 2010), while others focus on reporting and replication (e.g., TIDieR) [17, 18]. For tools developed for use within evidence-based medicine, the focus is often on whether systematic review findings can be applied to a specified context or setting [9]. Table 2 presents examples of this type of tool and includes the seminal work by Dans’ 1998 checklist in User’s Guides to the Medical Literature (intended for clinician’s use) and the tool developed by Atkins and colleagues for the Agency for Healthcare and Research Quality (intended for systematic review authors) [19, 20]. These types of tools include questions or criteria that prompt the clinician or review author to consider whether there are differences between the settings in the included studies and the setting in which the findings will be used.

**Table 2:** Examples of existing checklists to assess transferability/applicability, etc.

Checklist (author, year)	Checklist criteria
Dans 1998	Issues [for clinicians to consider when applying study findings to their context] Biologic (1) Are there pathophysiologic differences in the illness under study that may lead to a diminished treatment response? (2) Are there patient differences that may diminish the treatment response? Social and economic (3) Are there important differences in patient compliance that may diminish the treatment response? (4) Are there important differences in provider compliance that may diminish the treatment response? Epidemiologic (5) Do my patients have comorbid conditions that significantly alter the potential benefits and risks of the treatment? (6) Are there important differences in untreated patients’ risk of adverse outcomes that might alter the efficiency of treatment?
Lavis 2009	The following five questions can guide how to assess whether the findings from a systematic review are applicable to a specific setting. 1. Were the studies included in a systematic review conducted in the same setting or were the findings consistent across settings or time periods? 2. Are there important differences in on-the-ground realities and constraints that might substantially alter the feasibility and acceptability of an option? 3. Are there important differences in health system arrangements that may mean an option could not work in the same way? 4. Are there important differences in the baseline conditions that might yield different absolute effects even if the relative effectiveness was the same? 5. What insights can be drawn about options, implementation, and monitoring and evaluation?

Reprinted from: Dans [19] and Lavis [55]

Naturally, there have also been a number of attempts to identify and assess existing tools or common elements among tools. We have identified four reviews in particular which have examined tools for assessing external validity [21], tools for assessing transferability of health education interventions [10], tools to examine the external validity of health research [22], and tools for assessing applicability of findings in systematic reviews of complex interventions [9]. Two of these reviews focus on developing a tool to support *decision makers* in assessing whether evidence from a single primary study setting can be used in the decision makers’ context. The third review by Burchett and colleagues [23] looked at all frameworks and tools to assess external validity, applicability, or transferability and concluded that a validated framework for assessing transferability and applicability would be useful. The fourth review by Burford and colleagues examined existing tools for assessing applicability and how they apply to reviews of complex interventions [9]. The paper presents a number of questions to guide review authors on assessing applicability of review findings to a specific setting as well as providing review authors with guidance on what type of review information could support these assessments. Their analysis was based on a sample of existing checklists rather than a systematic search [9].

Research to develop existing tools and reviews of such tool suggest that systematic reviews include more information to help the end user consider transferability [9, 10, 24]. Such work should focus specifically on identifying factors influencing transferability and methods for assessing transferability [10], as well as guidance for how to gather information regarding the context of included studies in a systematic review that could aid in assessing applicability [9].

The current paper attempts to address some of these areas for future research by identifying (a) factors influencing transferability, (b) methods for assessing transferability, and/or (c) guidance for review authors on how to consider transferability through a systematic mapping and content analysis of existing tools and checklists for assessing transferability or related concepts. This review differs from previous reviews in that it (a) comes from the perspective of conducting systematic reviews to inform decision making in health care and social welfare and (b) aims to systematically identify the range of factors considered to influence transferability by examining the content of existing tools. Tools intended for primary study authors, systematic review authors, and decision makers are all included in an attempt to capture the full range of factors considered potentially important to the transferability of research findings (primary or secondary) as represented in the current literature.

### Aim

The aim of this study was (1) to systematically identify existing checklists and tools intended to address transferability or related concepts including applicability, generalizability, external validity, relevance, and transportability and (2) to develop a comprehensive overview of the criteria included in these checklists and tools. The ultimate objectives of this study are to (1) identify a checklist that supports review authors in considering transferability and (2) present a comprehensive list of factors to consider when assessing transferability of research, as described in existing tools. The output from the current review is intended to provide the basis for an approach that aims to systematically and transparently assess the transferability of review findings in order to improve the usefulness of systematic reviews for decision makers.

## Methods

### Eligibility criteria

We included papers (journal articles, guidance for research institutes, chapters from books, dissertations, etc.) that described a checklist to assess transferability (or associated terms, see discussion above) in primary or secondary and qualitative or quantitative research, including journal guidelines/instructions for authors, and information for practitioners. The checklists had to include criteria that were intended to be applied to a piece of primary or secondary research. We included articles if they were available in English, French, Spanish, Norwegian, Danish, or Swedish (determined by the language skills of the research team). Potentially relevant studies that were identified, but published in other languages are included in a list in Additional file 1.

We excluded articles where there were no checklist criteria, but rather a discussion of transferability or related concepts. Papers that discussed transferability and issues which could influence transferability at a theoretical level were not included. We also excluded checklists where transferability was not the main focus (e.g., critical appraisal tools that include one or two questions such as *Is this relevant for your population?*) and articles that described a list of strategies to improve transferability of research but that did not provide a checklist. Finally, we excluded checklists that were intended for assessing the applicability of guidelines to a local context, as we were only focusing on checklists that were intended to be used on primary or secondary research articles [25].

### Search strategy

An information specialist designed and conducted a systematic search of the literature in June 2016 without limitations to publication type, status, language, or date, to identify existing checklists or tools that examine transferability (hereafter used to refer to all related concepts, including applicability, generalizability, external validity).

We defined a checklist as a set of criteria to be used by a reader in evaluating the transferability of a piece of research. We searched eight databases (CINAHL, Cochrane Library, Embase, Epistemonikos, MEDLINE, PsycINFO). We also searched Proquest and Web of Science using similar terms. Search terms included modified versions of the terms “transferability,” “applicability,” “generalizability,” “external validity,” “in/directness,” or “feasibility”, and “checklist,” “prompt,” “guidance,” “guideline,” “tool,” “framework,” “evaluation study,” or “criteria” (see Additional file 2 for the full search strategy). In addition to a systematic search, we contacted experts, searched the reference lists of relevant publications, and conducted a gray literature search in Google Scholar using the terms “applicability” or “transferability” combined with “checklist” or “tool.” Finally, we searched the online repository of checklists managed by the EQUATOR network using the terms “transferability,” “applicability,” “generalizability,” and “external validity” (www.equator-network.org).

### Study selection

Using a web-based tool, Covidence, two review authors screened the titles and abstracts of references identified in the literature search [26]. Where there was disagreement, we promoted the reference to full-text screening. Two review authors screened potentially relevant records in full text according to the inclusion criteria described above. Disagreement was solved by discussion until consensus was reached.

### Data extraction process

We extracted data related to publication characteristics (title, author, language, country of first author) and checklist characteristics. The latter included the name of the checklist, the intended audience (e.g., researcher, peer reviewer, etc.), the study design to which the checklist could be applied (e.g., qualitative, quantitative), and the methods for developing the checklist (literature search, empirical evidence). Finally, we extracted the criteria included in the checklist, which were often presented as questions or prompts. Given that one of the ultimate objectives of this study is to identify guidance for review authors on assessing transferability, we extracted additional information from checklists intended to be used in the context of systematic reviews, specifically information on how the checklists were intended to be used (guidance for review authors) when this was described. Any guidance for review authors external to the actual checklist criteria for assessing transferability was not included in the content analysis, but is reported as part of the description of the checklist.

### Synthesis methods

We conducted a content analysis of criteria included in identified checklists using an inductive approach [27]. We extracted criteria from each checklist and then coded each criterion until we developed a set of categories. This was an iterative process, and we went several times to each checklist and re-coded criterion using the most current set of categories until we ended up with a final set of categories of criteria reported in this article. The codes were generated as we read through the checklists (e.g., “population demographic characteristics,” “political acceptability”). Some of the checklist criteria were vague or unexplained. When this was the case, we interpreted the criteria to the best of their ability and ensured there was a mutual understanding of the criteria between authors before coding the criteria into a “category of checklist criteria.” Finally, we sorted “categories of checklist criteria” into “themes.” We then conducted a frequency count for each category of checklist criteria by counting how many of the identified checklists included one or more criteria under each category [28].

## Results

**Fig. 1:**
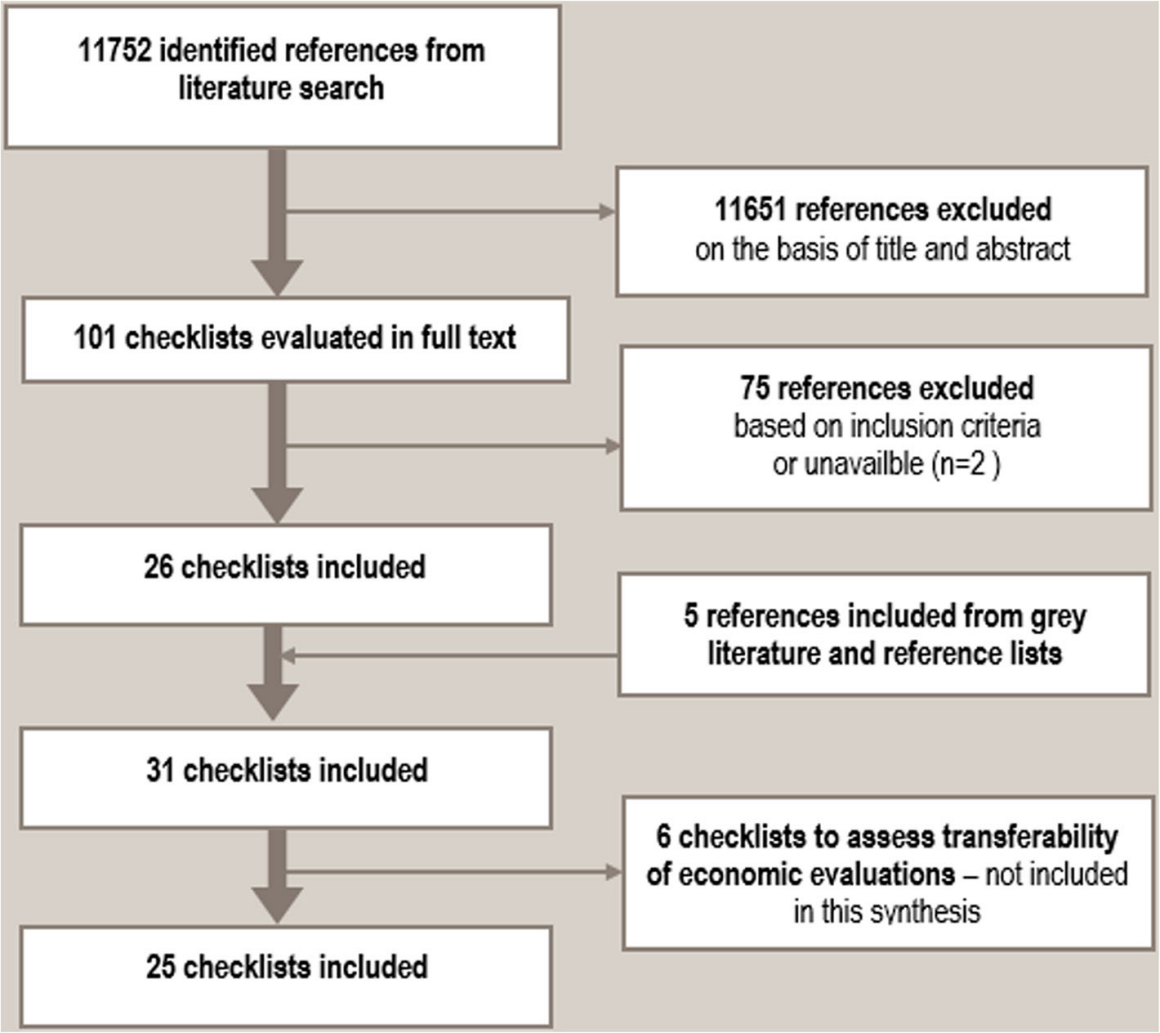
PRISMA flow diagram

We identified 11,752 references and read 101 articles in full text (see the Preferred Reporting Items for Systematic Reviews and Meta-Analyses diagram presented in Fig. 1; PRISMA flow diagram). Twenty-six of these articles described checklists that met our inclusion criteria. The other 75 publications were excluded because either did not describe a checklist, they described a checklist focused on something other than transferability, they were written in a language not included in our review, or the publication described a checklist that was not yet available and still under development. Through reference checking, we identified an additional five relevant checklists. In total, we identified 31 relevant checklists. Of these, six articles presented checklists to assess transferability of economic evaluations [28–33]. While relevant to systematic reviews, we considered checklists to assess transferability of economic evaluations to be substantially different from other types of identified checklists since they support checklist users to examine the costs associated with an intervention and not whether, or which, characteristics of the setting or population would influence the level of effectiveness (or perceptions and experiences) of the intervention in a specific context. These checklists were therefore not included in this analysis. This report is based on an analysis of the remaining 25 included checklists [8, 9, 11, 17–20, 24, 34–50].

### Characteristics of included checklists

The 25 included checklists were published between 1998 and 2016; however, only five were published after 2010. The checklists vary greatly according to aim (terminology used) and intended end user (who should use the checklist). With respect to terminology, the papers describing the checklists state that they are intended to assess applicability (*N* = 6), external validity (*N* = 5), generalizability (*N* = 4), transferability (*N* = 3), directness (1), replicability (*N* = 1), transportability (*N* = 1), implementation (*N* = 1), or a combination of applicability and either transferability or generalizability (*N* = 3). See Table 3 for an overview of included studies describing checklists.

**Table 3:** Overview of included studies describing checklists

Authors, year (ref)	Country of first author	Intended end user	Term used to describe checklist aim	Theme
Atkins 2010 [20]	USA	Review authors	Applicability	Health
Bonell 2006 [24]	UK	Primary study authors	Generalizability	Health
Bornhoft 2006	Germany	Primary study authors	External validity	Health
Buffett 2007 [36]	Canada	Decision makers	Applicability and transferability	Health
Burford 2013 [9]	Australia	Researchers and decision makers	Applicability	Health
Cambon 2013 [37]	France	Researchers and decision makers	Transferability	Health
Cuijpers 2005 [38]	Netherlands	Researchers and decision makers	Generalizability	Health
Currow 2009 [39]	Australia	Decision makers	Generalizability	Health
Dans 1998 [19]	Philippines	Clinicians	Applicability	Health
Dekkers 2010 [17]	Netherlands	Researchers and decision makers	External validity	Health
Feldstein 2008 [40]	USA	Researchers and decision makers	Implementation	Health
Glasgow 1999 [41]	USA	Primary study authors	Transferability	Health
Green 2006 [42]	USA	Clinicians	Relevance, applicability, generalization	Health
Gruen 2005 [43]	Australia	Review authors	Generalizability	Health
Hoffman 2014 [18]	Australia	Primary study authors	Replicability	Health
Horne 2016 [44]	USA	Decision makers	External validity	Social sciences
Lavis 2009 [45]	Canada	Decision makers	Applicability	Health
NHMRC 2000	Australia	Decision makers	Applicability	Health
Rothwell 2005 [46]	UK	Clinicians	External validity	Health
Rundall 2007 [47]	USA	Decision makers	Applicability	Health
Rychetnic 2002 [48]	Australia	Clinicians	Transferability	Health
Schoenwald 2001 [49]	USA	Clinicians	Transportability	Health
Schunemann 2013 [11]	Canada	Review authors	Indirectness	Health
Taylor 2007 [50]	Northern Ireland	Review authors	External validity	Social sciences
Wang 2006 [8]	Australia	Decision makers	Applicability and transferability	Health

The descriptions of the included checklists suggested that five checklists were primarily intended to be used by practitioners [19, 42, 46, 48, 49], seven by decision makers (this could include practitioners, as well as program managers, policy makers, politicians, etc.) [8, 34, 36, 39, 44, 45, 47], four by primary researchers (to presumably improve conduct and/or reporting) [18, 24, 35, 41], and five of the checklists were aimed at assisting both decision makers and researchers in making assessments [9, 17, 37, 38, 40]. Four of the identified checklists were intended for systematic review authors [11, 20, 43, 50] (see more details regarding these checklists in Table 3). Of the four checklists for review authors, one was intended to examine external validity of included studies [50]. Two were intended to be applied at the end of the systematic review in order to examine generalizability or indirectness of the review findings [11, 43]. The fourth checklist was intended to support authors of systematic reviews of effectiveness in health care to consider applicability throughout the systematic review [20]. See Table 4 for a detailed description of checklists intended to be used in the context of a systematic review.

**Table 4:** Overview of checklists for use by review authors

Author	Type of publication	Aim of checklist	Accompanying guidance on how to use the checklist	Stage to be used in systematic review process
Atkins 2010 [20]	Part of a methods guide for effectiveness reviews	To outline steps in “assessing and reporting applicability.”	1. Determine the most important factors that may affect applicability [criteria from corresponding checklist included in content analysis here] 2. Systematically abstract and report key characteristics that may affect applicability in evidence tables, highlight any effectiveness studies 3. Make and report judgments about major limitations to applicability of individual studies 4. Consider and summarize the applicability of a body of evidence	Throughout the systematic review process
Gruen 2005 [43]	Letter to the editor	“Generalizability [in a systematic review] can be tackled by considering the following questions…”	Not described	Not described
Schunemann 2013 [11]	Journal article	“to offer guidance to review authors tackling the challenge of judging the directness of evidence about review questions assembled in a systematic review[…]” This framework is intended to support and guide use of non-randomized controlled trials in systematic reviews on the effects of interventions.	“First, review authors should specify the PICO healthcare question that they are interested in addressing, defining the elements of the question in sufficient detail to facilitate judgments about directness. They can use the items in the subdomains and domains of Table 1 to specify their question as narrowly as necessary and as broadly as acceptable.…Second review authors should judge the directness of the evidence that they obtain on the basis of the factors in Table 1 [criteria included in content analysis in this systematic mapping]”	When developing the review question, and when applying GRADE to the review findings.
Taylor 2007 [50]	Journal article	“[…] the aim was to develop an approach that encompassed research into processes as well as studies of interventions, and that embraced a wider range of aspects of validity than the traditional Hierarchy of Evidence. Rather than seeking one hierarchy to cover all aspects, we sought to begin to develop a range of tools to appraise specific aspects of research design and methods.” Tools to appraise generalizability is one of five tools included in the range of tools described above.	The *Tools to appraise generalizability* is part of a set of five scales to appraise studies included in a systematic review. Studies were scored on each scale and the score was used to determine inclusion/exclusion in the review.	After studies have been identified that meet inclusion criteria.

The majority of the 25 checklists were developed for use in health research (*N* = 23). However, two checklists were developed for assessing research on interventions within the social sciences [44, 50]. All of the included checklists were aimed at assessing effectiveness research (quantitative data). Seven of the checklists were published by research groups from Australia, seven from the USA, and the rest from Canada (3), UK (2), Netherlands (2), Germany (1), France (1), Northern Ireland (1), and Philippines (1).

### Results of content analysis

The results of the content analysis are based on an analysis of individual checklist criteria from the 25 included checklists. Many of the checklist criteria we identified were written in the form of a key question followed by supporting questions. For example, the checklist reported in Wang 2005 includes the following item:

Are the characteristics of the target population comparable between the study setting and the local setting? With regard to the particular aspects that will be addressed in the intervention, is it possible that the characteristics of the target population, such as ethnicity, socioeconomic status, educational level, etc will have an impact on the effectiveness of the intervention? [8]

We have chosen to focus on the checklist key questions or items (e.g., “Are the characteristics of the target population comparable between the study setting and the local setting?” [9]). However, in some cases we have also extracted data from the supporting questions, for example, when the supporting questions discuss unique and specific issues (e.g., educational level), or when we interpreted a supporting question as being related to something other than the key question for which it was intended to illustrate. Since we have not “counted” the number of times something was mentioned, but rather the number of studies that include each specific criteria, we assume that neither splitting nor double-coding of criteria is problematic for this analysis. Furthermore, this content analysis is based on items from the included checklist, whether or not it is our personal opinion that these items/factors/criteria are related to transferability. Therefore we have coded all included checklist criteria, although some may not appear to be directly related to transferability or related concepts.

Through the content analysis we identified seven themes: *population, intervention, implementation context, comparison condition, outcomes, environmental context* and *researcher conduct*. Under each theme we have identified categories of checklist criteria. For the theme *intervention*, we grouped categories of criteria into one of two subthemes: *intervention characteristics*, *intervention delivery*. For the theme *implementation context (immediate)* we grouped categories of checklist criteria into two subthemes: *service providers (individuals)* and *implementing organization*. The themes and categories of criteria are described below and presented in detail in Table 5.

**Table 5:** Themes, categories and transferability factors identified in content analysis of included checklists

Theme	Subtheme	Category of criteria	Number of studies (*N*)
Population		Participant characteristics	20
		Characteristics of illness (description of condition and comorbidities, other risk for adverse effects)	8
		The acceptability of the intervention to the participants	4
		Source of referral (where patients/clients are referred from, e.g., specialist or general practice)	2
		Participants’ preferences regarding the intervention	2
		Participant need for/access to information about the intervention	2
		Availability of personal support for participants	1
		Participants’ exposure to other interventions or previous exposure to current intervention	1
		Participant compliance	1
		Participant satisfaction with the intervention	1
Intervention	Intervention characteristics	Intervention design (complexity and clarity)	5
		Intervention theory	4
		Category of intervention (policy, practice, program, guideline)	2
		Name of the intervention	1
	Intervention delivery	Can the intervention be tailored for different settings?	5
		How often/intensely was the intervention delivered? (Frequency/intensity)	4
		In which settings was the intervention delivered? (physical setting, etc.)	3
		How long the intervention was implemented? (duration)	2
		What materials/manuals were used to deliver the intervention?	2
		Standard procedures for the intervention in a real life setting?	2
		Intervention delivery details (generally)	1
		Who pays for the intervention?	1
Implementation context (immediate)	Service providers (individuals)	Skills of service providers	8
		Training of service providers	6
		Type of service provider	5
		Service provider characteristics	2
		Monitoring and supervision of service providers	2
		Factors that affect motivation of service providers	2
		Service provider compliance	1
		Number of service providers	1
	Implementing organization	Essential resources (e.g., financial, human, material resources for development, testing, implementation and recruiting)	9
		Culture of the implementing organization (e.g., missions, mandates, climate, readiness for implementation)	6
		Size and structure of the implementing organization	5
		Organizational policies (e.g., administrative, personnel, hierarchies)	3
		Implementing organization—interagency working relationships	2
		Implementing organization—financing methods	1
		Implementing organization level or specialty of care	1
		Motivation of implementing organization	1
		Identification of implementing organization	1
		Communication regarding implementation	1
		Endorsement of the intervention	1
		Ease of trial implementation (ability to do a small scale introduction of the intervention)	1
		Is it feasible for the implementing organization to implement the intervention?	7
		How does the intervention work over time (e.g., Evolution/sustainability of intervention)	3
		Implementation fidelity (consistency of intervention delivery across staff and intervention components, consider process evaluations)	5
		Support for implementing the intervention	1
Comparison intervention		Characteristics of usual services	2
		Quality of comparison intervention	1
		Type of comparison condition	1
		Skills of service providers for comparison condition	1
		Duration of comparison condition	1
		Interventions accompanying comparison condition	1
		Procedures for implementing comparison intervention	1
Outcomes		Key outcomes are considered, including those that are important to the client/patient	6
		Adverse effects are considered	4
		Costs associated with intervention	3
		Details of follow-up period	4
		Organizational/societal level outcomes	3
		How are outcomes measured	3
		Sensitivity analyses conducted	2
		Consistency of findings	1
		Surrogate outcomes are used	2
Environmental context		Temporal context (e.g., if the intervention has changed over time)	2
		Regulatory context (local regulation or legislature)	2
		Political context (political acceptability)	5
		Systems context (Health systems arrangements)	6
		Community need (baseline prevalence/risk status)	12
		Social acceptability at community level	6
		Social context generally (including racial/ethnic issues)	3
		Local professional/expert opinion	1
		Alternative interventions offered at the same time	4
		Co-interventions offered to/necessary for participants	1
		Physical or geographic setting	9
Researcher conduct		Participation rate	5
		How participants were selected	5
		Eligibility criteria of participants in a study	4
		Length and details of the run-in period	3
		How participants were recruited	1

Four of the themes are reflective of the standard way of formulating a systematic review question (population, intervention, comparison, outcome; PICO). Many of the categories under these themes would often be considered when a primary researcher or systematic review author formulates a research question related to the effectiveness of an intervention. The other three themes relate to either context or researcher conduct and will be discussed in more detail below.

### Theme: Population

This theme describes categories of criteria related to characteristics of the population. Characteristics of the population in this case is conceptualized quite broadly and includes not only the demographic characteristics of the population, but also characteristics of the participants condition/illness, the acceptability of the intervention to the participants and/or their preferences, from where or how the participants were referred to the intervention, participants’ need for/access to information about the intervention, availability of personal support for participants, participants’ exposure to other interventions or previous exposure to current intervention, participants’ compliance and/or satisfaction with the intervention. The majority of the included checklists included one or more checklist criterion that were coded into one or more of the categories under the theme *Population* (*N* = 20; [8, 9, 11, 17, 19, 20, 24, 34–40, 42, 44, 46–49]). These criteria were described using different terms. General terms such as *demographic characteristics* or *population characteristics* were often used. However, some checklists included more specific questions related to ethnicity, socio-economic aspects, age, workforce participation, and education. Approximately one third of the checklists (*N* = 8) included some criteria related to characteristics of the participants’ illness or condition, including comorbidities [11, 17, 19, 20, 34, 35, 39, 46] . In four checklists, criteria related to patient acceptability were included [8, 9, 36, 48]. Only one or two checklists included items that were coded into the other categories under the theme *Population* (see Table 5 for details).

### Theme: Intervention

This theme describes categories of criteria directly related to the intervention. Many of these themes appeared to be interrelated, and so we grouped these categories into subthemes, “intervention characteristics”, and “intervention delivery”, described below.

#### Subtheme: Intervention characteristics

The subtheme *intervention characteristics* describes the categories of criteria related to the name of the intervention being examined, the complexity and clarity of the intervention design, the theory supporting the intervention, or whether the intervention is policy, practice, program or guideline could influence the transferability of the intervention. Less than one third of checklists (*N* = 8) included criteria that were coded into one or more of these categories [11, 18, 24, 40, 42, 47–49].

#### Subtheme: Intervention delivery

The subtheme *intervention delivery* describes the categories of criteria related to how an intervention is (intended to be) delivered. These categories describe checklist criteria that ask end users to consider the following issues when assessing transferability or related concepts: whether there is a possibility for tailoring an intervention, the intensity and duration of an intervention, the materials/manuals used to deliver an intervention and the settings in which the intervention is delivered (hospital, home, etc.). Some categories describe criteria that ask users of a checklist to consider whether there are standard procedures for the intervention in a real life setting, who pays for the intervention and whether any other general details related to intervention delivery could influence transferability. Twelve checklists included criteria that were coded into one or more categories under this subtheme [8, 9, 11, 17, 18, 35, 39, 40, 44, 46, 48, 49].

### Theme: Implementation context (immediate)

This theme describes categories of criteria that are external to the intervention, but that influence how the intervention is delivered/received. The difference between this theme and the subtheme *Intervention delivery* described above is that the categories of criteria included under this theme are not related to the design or description of an intervention. While the subtheme *intervention delivery* is intended to cover categories of criteria related to intervention delivery itself, categories of criteria under this theme (*implementation context (immediate)*) relate to ways in which people and organizations may influence the implementation of an intervention. Under this theme, we grouped categories into two subthemes: *individual service providers* and *implementing organizations*.

#### Subtheme: Individual service providers

This subtheme describes categories of criteria related to individual service providers responsible for providing an intervention. These categories include considering how the number, and type, of service provider(s) are responsible for implementing the intervention, and their skills, training, and other characteristics could influence transferability. Other categories in this subtheme are related to practices around monitoring and supervision of the service providers, their compliance, and factors that influence the motivation of service providers. Sixteen checklists included criteria that were coded into the categories of criteria under this subtheme [8, 9, 17–20, 37–42, 44, 48–50].

#### Subtheme: Implementing organizations

This subtheme describes categories of criteria that are related to the organization responsible for implementing an intervention. Categories of criteria under this theme include the amount of essential resources that are available to the implementing organization, (e.g., financial, human, material resources for development, testing, implementation and recruiting), the culture of the implementing organization with respect to the organizational missions, mandates, climate, and how ready the organization is for implementation, and whether it is feasible for the organization to implement the intervention. Other categories describe criteria related to more practical issues such as the size and structure of the implementing organization. Six checklists included criteria that were coded into one or more of the categories under this subtheme [8, 9, 36, 41, 44, 49]. We present a full list of the categories of criteria included in this subtheme in Table 5.

### Theme: Comparison intervention

This theme describes categories of criteria related to the comparison condition. Only four checklists included criteria related to the comparison intervention, and these criteria were coded into one of the following categories: characteristics of the comparison condition and quality of the usual services to which an interventions effect is being compared [9, 11, 20, 35]. Three of the checklists that included criteria that were coded into categories under this theme are for systematic review authors [9, 11]*.*

### Theme: Outcomes

This theme describes categories of criteria related to the outcomes on which an intervention aims to influence. Nine checklists included criteria that were coded into the following categories under the theme “outcomes”: how the outcome was measured, length of follow-up, key outcomes, or adverse outcomes [11, 20, 39–42, 45, 46].

### Theme: Environmental context

This theme describes categories of criteria that go beyond the immediate implementation setting (e.g., service providers or implementing organization). The following categories describe criteria that asks the end user to consider issues related to: temporal context (i.e., if the intervention has changed significantly over time so that a study from 2000 would describe an intervention very different than one from 2010); political context (political acceptability, governing system, etc.); regulatory context (how the intervention fits with existing legislation); systems context (organization of health or welfare care, employment regulations or practices); social context (social cohesion, levels of community trust, presence of racism); other interventions (an environment where multiple competing interventions are implemented concurrently, or where one intervention is closely related to participation in another intervention), and; the geographic or physical setting. Twenty checklists included criteria related to one or more of these categories [8, 9, 11, 17, 20, 24, 34–40, 43–49].

### Theme: Researcher conduct

This theme describes categories related to how researcher conduct may influence transferability. The categories primarily include criteria that are related to issues within the control of the research team investigating the intervention, such as participation rate, how participants were selected, eligibility criteria of participants in a study, length and details of the run-in period and how participants were recruited. In total, eight identified checklists included criteria that were coded into these categories as factors under this theme. Five of these checklists were aimed at assessing external validity [17, 35, 44, 46, 50], however, one checklist aimed to examine transferability [41], one looked at applicability [20] and one aimed to assess “relevance, applicability and generalizability” [42]. The checklists which included criteria under this theme also included criteria related to categories under the other themes, and were thus not solely concerned with the influence of researcher conduct on transferability or related concepts.

## Discussion

The mapping component of this project resulted in 25 checklists to assess transferability and related concepts. Only four of these checklists were aimed at systematic review authors, and only two of these were published in the last decade. The criteria for inclusion in this mapping was very restrictive (only checklists with explicit criteria) which means that there may be many more guidelines or discussions of issues to consider related to transferability that we did not include. Regardless, it was surprising given the current focus on making systematic review findings relevant to decision makers, that not more checklists and tools are available for systematic review authors to support this work [9]. Furthermore, with the increasing use of qualitative evidence to inform decision making, it was surprising that none of the included checklists appear to be aimed at assessing transferability of qualitative research [16]. This could be because there is less emphasis on transferability in the qualitative discipline and more of a focus on understanding of individual motivation, experiences, and mechanisms [16, 51].

The content analysis produced seven themes of concepts (and subcategories of concepts) that describe checklist criteria end users should consider in their assessment of transferability or related concepts. Four of these categories are related to a typical systematic review question formulation (PICO) which is likely the result of the authors’ familiarity with this research method. Two of the remaining themes described concepts (checklist criteria) related to contextual issues that cut across population, intervention, comparison and outcomes. The final category, related to researcher conduct, is not considered important for the purpose of this review; however, it does illustrate the breadth of concepts that could be imagined to influence transferability and related concepts.

### Terminology

In this project we purposely included a broad range of checklists with respect to their stated aims, the terminology used and the intended audience. Since we were first and foremost interested in identifying a checklist that provides sufficient guidance for review authors on how to consider transferability in a systematic review, and thereafter interested in identifying all factors imagined to potentially influence transferability, we deemed it necessary to include any checklist that aimed to assess one of the concepts related to transferability. Given the lack of consensus on terminology regarding concepts related to transferability, it was conceivable that a potentially relevant checklist may be described as intended for assessing applicability, generalizability, relevance, etc. The results of the content analysis provide support for this hypothesis, in that there appears to be little to no consistency between the aim of the checklist (terminology) and the type of checklist criteria that the checklist included. For example, among the 16 checklists that included criteria related to the subcategory “individual service providers,” were checklists intended to assess transferability, applicability, replicability, generalizability, implementation, relevance, transportability and external validity [8, 9, 17–20, 37–42, 44, 48–50]. Checklists seemingly intended to assess different concepts include broadly similar criteria. Furthermore, there was overlap in which criteria was included in checklists that were described as assessing different concepts. Eight checklists included one or more criterion related to concepts under the theme *Researcher conduct.* Most of the concepts included in this theme are traditionally associated with external validity. However, three of the checklists that included criteria related to this theme aimed to assess applicability, transferability and “relevance, applicability and generalizability” [17, 20, 35, 41, 42, 44, 46]. That checklists explicitly aimed at assessing applicability, transferability and “relevance, applicability, and generalizability” include criteria common to other checklists explicitly aimed at assessing external validity is a good example of the confusion regarding terminology and concepts related to transferability, and the fuzziness of barriers between these related concepts.

Moreover, there appeared to be no consistency between the terminology used to describe the aim of a checklist and the intended end user of the checklist. Specifically, different terminology was used to describe the aim of checklists that appeared to target the same end user (e.g., practitioner) within the same discipline (e.g., health). Among the six checklists aimed at assisting decision makers within health, three checklists used the term “applicability,” two used “applicability and transferability” and one used “generalizability” [8, 34, 36, 39, 45, 47].

Finally, we identified a number of “new” terms in the included checklist that we were not previously aware of as being used to describe issues related to transferability, applicability, etc., such as *replicability* and *transportability*. Future attempts to systematically map these types of checklists, or literature discussing transferability, etc., may include these terms in their search strategies.

### Focus on health care

The majority of the identified checklists were intended to be used within health research, which is not surprising given the databases included in the systematic search. However, that the overwhelming work done on assessing concepts related to transferability has been done in health research may have consequences. For instance, one surprising result of the content analysis is that none of the identified checklists included factors related to religion, family structure, social equality, or welfare services. Within social care and public health, such factors could be considered important to the transferability of some review findings. However, even the two checklists intended for use in social science research did not mention these types of issues. There is a clear lack of checklists intended to identify and assess factors that could influence transferability across a range of health *and* social care interventions.

None of the included checklists, thus, were adequate in addressing our initial aim of identifying a checklist to support systematic review authors in considering, and assessing, transferability of review findings to the context of interest in the review. On first glance, the checklist developed by Atkins and colleagues for the Agency for Healthcare and Research Quality (AHRQ) was the most comprehensive of those identified from the perspective of conducting systematic reviews [20]. However, we identified two important limitations of this checklist for our purposes: a lack of detailed guidance on how to actually perform the assessment of *applicability*, and that it is explicitly intended to be used in systematic reviews of effectiveness for health care, and not in other types of systematic reviews or for social care or public health interventions.

### Previous reviews

We attempted to compare the results of our content analysis to the three previous reviews of checklists and tools for assessing external validity [21], transferability in health education interventions [10] and external validity, transferability and applicability in health research [22].

### Limitations and future research

The project was undertaken from the perspective of systematic review authors, and may be influenced by our experience in conducting systematic reviews of population level interventions, and engaging directly with commissioners of systematic reviews. Specifically, we were inclined to categorize according to the most commonly used template of forming review questions (PICO; population, intervention, comparison and outcome characteristics), rather than other templates such as PICOS (population, intervention, comparison, outcome, study design), PIPOH (population, interventions, professionals, outcomes, health care setting) or templates used to formulate questions in qualitative evidence syntheses (e.g., SPICE; setting, perspective, phenomenon of interest, comparison, evaluation) [52, 53]. We are also aware that our analysis may have been influenced by feedback we have previously received from stakeholders whom we have worked with on previous projects and their perception of transferability factors.

We chose to exclude checklists assessing transferability of economic evaluations after reading them and noting that they were substantively different than the included checklists. This is potentially a limitation of our analysis since some of the themes captured by such checklists are undoubtedly relevant in many systematic reviews. However, we deemed these checklists to be outside the scope of the current paper.

The content analysis is based on an interpretation of the checklist criteria, questions, and supporting questions included in existing checklists. The analysis does not include information presented in the included checklists regarding the theoretical underpinnings of specific checklist criteria. Thus where the checklist item is summarized simply in one or two words, the interpretation of the checklist item’s meaning is via the lens of a systematic review author, and therefore may not always match the checklist authors’ intention when including said item.

Future work to examine terminology related to transferability should focus on whether terminology is related to purpose. In other words, do researchers or clinicians set different standards or expectations when a checklist is aimed at assessing transferability versus applicability, or generalizability?

Due to the current lack of consensus regarding terminology related to transferability, the systematic literature search may have missed relevant checklists. We attempted to account for this by checking reference lists of key articles and consulting methodological experts. Furthermore, given the plethora of checklists identified, it is conceivable that many other checklists exist in languages other than the languages included in this review and are for local use within an institution. A further consequence of confusions regarding terminology for this systematic mapping is the possible inclusion of checklists that assess concepts related to transferability, but which have a considerably different focus. For instance, some of the identified checklists examine external validity, and while this term is generally thought to be related to the concept of transferability, the factors on which one should base an assessment of external validity differ substantially from those we would imagine as potentially important in assessing transferability. The result of the content analysis is thus a long list of factors that vary greatly in how relevant that are to assessing transferability of research findings. This systematic mapping and content analysis will be used to inform the development of a structured conversation guide to be used to discuss transferability with decision makers in a systematic review process.

## Conclusions

We identified 25 checklists that are intended to help practitioners, decision makers, researchers or review authors assess transferability, applicability, generalizability, external validity, relevance, or transportability. Through the content analysis of the individual criteria included in these checklists, we developed a list of seven themes of categories of criteria for end users to consider in making an assessment of transferability. We believe that this list is comprehensive and can serve as a basis in developing guidance for review authors on how to systematically and transparently consider transferability in a systematic review process [54].

## Additional files

Additional file 1: Additional checklists not included in the analysis. (DOCX 16 kb)

Additional file 2: Search strategy. (DOCX 18 kb)

## Abbreviations

AHRQ: Agency for Healthcare and Research Quality
CINAHL: The Cumulative Index to Nursing and Allied Health Literature database
EQUATOR: Enhancing the QUAlity and Transparency Of health Research
GRADE: Grading of Recommendations Assessment, Development, and Evaluation
GRADE-CERQual: Approach for assessing Confidence in Evidence from Reviews of Qualitative research
PICO: Population, Intervention, Comparison, Outcome
PICOS: Population, Intervention, Comparison, Outcome, Study design
PIPOH: Population, Interventions, Professionals, Outcomes, Health care setting
PRISMA: Preferred Reporting Items for Systematic Reviews and Meta-Analyses
SPICE: Setting, Perspective, phenomenon of Interest, Comparison, Evaluation
TIDieR: The Template for Intervention Description and Replication Checklist
