# Supplementary material for: Systematic mapping of checklists for assessing transferability
Source: Syst Rev. 2019 Jan 14;8:22. doi: 10.1186/s13643-018-0893-4 (PMC6330740; doi:10.1186/s13643-018-0893-4)
Supplement: Supplementary file 1 — Additional checklists not included in the analysis. (DOCX 16 kb) [file 13643_2018_893_MOESM1_ESM.docx]

**Additional file 1: Additional checklists not included in the analysis**

### Checklists published in other languages

Milano M, Collecchia G: **Transferability of results from the literature to general practice: An evaluation chart. [Italian]**. *Ricerca e Pratica* 2002, **18**(5):211-216.

### Checklists – under development

Loudon K, Zwarenstein M, Sullivan F, Donnan P, Treweek S: **PRECIS-2: A tool to improve the applicability of randomised controlled trials**. *Trials* 2013, **14**:31.

### Checklists assessing transferability of economic evaluations

Antonanzas F, Rodriguez-Ibeas R, Juarez C, Hutter F, Lorente R, Pinillos M: **Transferability indices for health economic evaluations: Methods and applications**. *Health Economics* 2009, **18**(6):629-643.

Boulenger S, Nixon J, Drummond M, Ulmann P, Rice S, de Pouvourville G: **Can economic evaluations be made more transferable?** *Eur J Health Econ* 2005, **6**(4):334-346.

Drummond M, Barbieri M, Cook J, Glick HA, Lis J, Malik F, Reed SD, Rutten F, Sculpher M, Severens J: **Transferability of economic evaluations across jurisdictions: ISPOR Good Research Practices Task Force report**. *Value Health* 2009, **12**(4):409-418.

Nixon J, Rice S, Drummond M, Boulenger S, Ulmann P, de Pouvourville G: **Guidelines for completing the EURONHEED transferability information checklists**. *Eur J Health Econ* 2009, **10**(2):157-165.

Späth H, Carrère M, Fervers B, Philip T: **Analysis of the eligibility of published economic evaluations for transfer to a given health care system: Methodological approach and application to the French health care system**. *Health Policy* 1999, **49**(3):161-177.

Welte R, Feenstra T, Jager H, Leidl R: **A decision chart for assessing and improving the transferability of economic evaluation results between countries**. *Pharmacoeconomics* 2004, **22**(13):857-876.
