# Supplementary material for: Systematic mapping of checklists for assessing transferability
Source: Syst Rev. 2019 Jan 14;8:22. doi: 10.1186/s13643-018-0893-4 (PMC6330740; doi:10.1186/s13643-018-0893-4)
Supplement: Supplementary file 2 — Search strategy. (DOCX 18 kb) [file 13643_2018_893_MOESM2_ESM.docx]

**Additional file 2: Search strategy**

### Database searches

**Database: (OVID) MEDLINE**

**Date: 06.06.2016**

**Hits: 5622**

| **#** | **Searches** | **Results** |
| --- | --- | --- |
| 1 | ((transferabilit* or applicabilit* or generali#abilit* or external validit* or directness* or indirectness* or feasibilit*) adj8 (Checklist* or check list* or prompt* or guidance* or guide? or guideline* or tool? or framework* or evaluation stud* or criteria)).tw. | 5952 |
| 2 | (transferabilit* or applicabilit* or generali#abilit* or external validit*).kw. | 225 |
| 3 | 1 or 2 | 6153 |
| 4 | exp Animals/ | 20329271 |
| 5 | Humans/ | 16068659 |
| 6 | 4 not (4 and 5) | 4260612 |
| 7 | 3 not 6 | 5737 |
| 8 | remove duplicates from 7 | 5622 |

**Database: (OVID) PsycInfo**

**Date: 06.06.2016**

**Hits: 4098**

| **#** | **Searches** | **Results** |
| --- | --- | --- |
| 1 | ((transferabilit* or applicabilit* or generali#abilit* or external validit* or directness* or indirectness* or feasibilit*) adj8 (Checklist* or check list* or prompt* or guidance* or guide? or guideline* or tool? or framework* or evaluation stud* or criteria)).tw. | 1613 |
| 2 | (transferabilit* or applicabilit* or generali#abilit* or external validit*).id. | 2637 |
| 3 | 1 or 2 | 4100 |
| 4 | remove duplicates from 3 | 4098 |

**Database: (OVID) EMBASE**

**Date: 06.06.2016**

**Hits: 696**

| **#** | **Searches** | **Results** |
| --- | --- | --- |
| 1 | ((transferabilit* or applicabilit* or generali#abilit* or external validit* or directness* or indirectness* or feasibilit*) adj8 (Checklist* or check list* or prompt* or guidance* or guide? or guideline* or tool? or framework* or evaluation stud* or criteria)).tw. | 7824 |
| 2 | (transferabilit* or applicabilit* or generali#abilit* or external validit*).kw. | 906 |
| 3 | 1 or 2 | 8653 |
| 4 | limit 3 to exclude medline journals | 783 |
| 5 | (abstract or conference or conference paper or conference proceeding or conference proceeding article or conference proceeding conference paper or conference proceeding editorial or conference proceeding note).pt. | 2973216 |
| 6 | 4 and 5 | 149 |
| 7 | 4 or 6 | 783 |
| 8 | limit 7 to embase | 772 |
| 9 | nonhuman/ | 4762573 |
| 10 | 8 not 9 | 699 |
| 11 | remove duplicates from 10 | 696 |

**Database: Cochrane Library**

**Date: 06.06.2016**

**Hits: 782**

Search 1

ID Search Hits

#1 ((transferabilit* or applicabilit* or generali?abilit* or external validit* or directness* or indirectness* or feasibilit*) near/7 (Checklist* or check list* or prompt* or guidance* or guide or guides or guideline* or tool or tools or framework* or evaluation stud* or criteria)):ti,ab,kw in Cochrane Reviews (Reviews and Protocols), Trials and Methods Studies 600

Search 2

ID Search Hits

#1 ((transferabilit* or applicabilit* or generali?abilit* or external validit* or directness* or indirectness* or feasibilit*) near/7 (Checklist* or check list* or prompt* or guidance* or guide or guides or guideline* or tool or tools or framework* or evaluation stud* or criteria)) in Other Reviews, Technology Assessments and Economic Evaluations 182

**Database: (EBSCO) CINAHL**

**Date: 06.06.2016**

**Hits: 256**

| S1 | TX ((transferabilit* or applicabilit* or generali#abilit* or "external validit*" or directness* or indirectness* or feasibilit*) N7 (Checklist* or "check list*" or prompt* or guidance* or guide# or guideline* or tool# or framework* or "evaluation stud*" or criteria)) | 1 040 |
| --- | --- | --- |
| S2 | MJ (transferabilit* or applicabilit* or generali#abilit* or "external validit*") | 75 |
| S3 | S1 OR S2 | 1 108 |
| S4 | S1 OR S2 [Limiters - exclude MEDLINE records] | 256 |

**Database: Epistemonikos**

**Date: 09.06.2016**

**Hits: 1166**

[Title or Abstract]: transferabilit* OR applicabilit* OR generalisabilit* OR generalizabilit* OR "external validity" OR directness* OR indirectness* OR feasibilit*

AND

[Title or Abstract]: checklist* OR "check list" OR prompt* OR guidance* OR "guide" OR "guides" OR guideline* OR "tool" OR "tools" OR framework* OR "evaluation study" OR criteria

### Other searches

**ProQuest Sociological Abstracts & Social Services Abstracts**

**Date: 06.06.2016**

**Hits: 290**

TI,AB,SU((transferabilit* or applicabilit* or generalisabilit* or generalizabilit* or "external validit*" or directness* or indirectness* or feasibilit*) NEAR/7 (checklist* or "check list*" or prompt* or guidance* or "guide" or "guides" or guideline* or "tool" or "tools" or framework* or "evaluation stud*" or "criteria"))

332 = 290 after de-duplication

**Web of Science Core Collection**

**Date: 06.06.2016**

**Hits: 877**

TS=((transferabilit* or applicabilit* or generali?abilit* or "external validit*" or directness* or indirectness* or feasibilit*) NEAR/7 (checklist* or "check list*" or prompt* or guidance* or "guide" or "guides" or guideline* or "tool" or "tools" or framework* or "evaluation stud*" or "criteria"))

8390

Refined by: WEB OF SCIENCE CATEGORIES: ( HEALTH CARE SCIENCES SERVICES OR PSYCHOLOGY MULTIDISCIPLINARY OR MULTIDISCIPLINARY SCIENCES OR PSYCHOLOGY OR EDUCATION SCIENTIFIC DISCIPLINES OR NURSING OR SOCIAL SCIENCES INTERDISCIPLINARY OR PSYCHOLOGY CLINICAL OR HEALTH POLICY SERVICES )

Timespan: All years. Indexes: SCI-EXPANDED, SSCI.

877

**Google Scholar**

**Date: 28 October 2016**

Search terms: Transferability AND checklist; Transferability AND tool; Applicability AND checklist; Applicability AND tool
